# Characterization of a Plastoglobule-Localized SOUL4 Heme-Binding Protein in *Arabidopsis thaliana*

**DOI:** 10.3389/fpls.2020.00002

**Published:** 2020-01-31

**Authors:** Venkatasalam Shanmugabalaji, Bernhard Grimm, Felix Kessler

**Affiliations:** ^1^Laboratory of Plant Physiology, Institute of Biology, University of Neuchâtel, Neuchâtel, Switzerland; ^2^Institute of Biology/Plant Physiology, Humboldt University Berlin, Berlin, Germany

**Keywords:** plastoglobule, SOUL4, heme-binding protein, chloroplast, kinase

## Abstract

Heme plays an active role in primary plant metabolic pathways as well as in stress signaling. In this study, we characterized the predicted heme-binding protein SOUL4. Proteomics evidence suggests that SOUL4 is a component of *Arabidopsis* plastoglobules (PGs, chloroplast lipid droplets). SOUL4 contains heme-binding motifs and the recombinant protein is shown here to bind heme *in vitro*. Fluorescence-tagged SOUL4 colocalized with the specific PG marker Fibrillin1A (FBN1A) in transiently transformed *Nicotiana benthamiana* leaves. In addition, SOUL4 cofractionated with another PG marker Fibrillin2 (FBN2) in sucrose gradient ultracentrifugation experiments. *In vitro* kinase experiments revealed that SOUL4 is phosphorylated by a yet unknown chloroplast protein kinase. Our data demonstrate that SOUL4 is a *bona fide* PG protein and may function in heme-buffering in the chloroplast.

## Introduction

Tetrapyrroles play key roles in photosynthesis, respiration, and in various other biological processes. In plants, chlorophyll, heme, and phytochromobilin (PΦB) are the main tetrapyrroles derived from a common biosynthetic pathway in chloroplasts (Mochizuki et al., 2010). Due to their photochemical properties, misregulation of tetrapyrrole metabolism may lead to severe photo-oxidative damage and cell death (Busch and Montgomery, 2015). Thus, tetrapyrrole transport and metabolism need to be tightly controlled (Brzezowski et al., 2015; Kobayashi and Masuda, 2016). Protoporphyrin IX serves as a common intermediate in both the chlorophyll- and heme-synthesized branches of tetrapyrrole metabolism. In the heme branch, ferrochelatase inserts Fe2+ into protoporphyrin IX to form heme. Heme is further degraded biliverdin IXα by heme oxygenase. In plants, biliverdin IXα can be reduced by phytochromobilin synthase to phytochromobilin, which is the chromophore of the phytochrome photoreceptor family (Mochizuki et al., 2010). The intracellular trafficking of heme from the chloroplasts to the cytosol, mitochondria, as well as other organelles remains to be elucidated. Oxidative stress and ABA perception lead to increased heme content in the cell (Vanhee et al., 2011). Accumulating heme molecules are capable of reacting with oxygen to generate cytotoxic ROS (Busch and Montgomery, 2015). To prevent this, a number of heme-binding proteins exist in various cellular compartments. It is proposed that these proteins protect the cell from the cytotoxic effects and bind to heme covalently and/or noncovalently (Balestrasse et al., 2005; Espinas et al., 2012).

Heme-binding proteins may act as carriers, transporters, or regulators of the remaining free heme pool. In plants, tryptophan-rich sensory protein (TSPO) is a well-known heme-binding protein present in most subcellular compartments (Veenman et al., 2016). Under oxidative stress, excess heme binds to TSPO and interacts with autophagy-related protein 8 (ATG8) for further degradation through the autophagy pathway (Vanhee et al., 2011). Furthermore, *Arabidopsis* SOUL heme–binding protein 5 (SOUL5) has been identified as a heme-binding protein that interacts with heme oxygenase in the chloroplast (Lee et al., 2012).

A first SOUL protein was reported in the chicken retina and pineal gland, specifically expressed in response to light signaling (Zylka and Reppert, 1999). The SOUL protein uses a His-residue as an axial ligand to coordinate the Fe(II)-containing heme. Heme-binding triggers the formation of hexameric SOUL complexes (Sato et al., 2004; Dias et al., 2006). Five SOUL isoforms have been annotated in *Chlamydomonas reinhardtii* (Merchant et al., 2007). The *Chlamydomonas* SOUL3 homolog has been implicated in the organization and positioning of the eyespot within the cell and may play a role in algal light perception (Schulze et al., 2013). In *Arabidopsis*, there are five proteins with a SOUL motif. *In vitro* studies demonstrated that SOUL1 (At1g17100) and SOUL2 (At2g37970) are cytosolic and bind to heme as well as other porphyrins (Takahashi et al., 2008). The proteome of the *Arabidopsis* chloroplasts revealed the presence of SOUL5 at the thylakoid membrane (Peltier et al., 2006) which was further confirmed in a localization study (Lee et al., 2012). The SOUL4 protein (At3g10130) was identified in the proteome of plastoglobules (PGs), another chloroplast subcompartment (Vidi et al., 2006; Ytterberg et al., 2006; Lundquist et al., 2012).

PGs are lipoprotein particles within the chloroplast. PGs are bounded by a membrane lipid monolayer that is contiguous with the outer leaflet of the thylakoid membrane. The interior space of PG is filled with hydrophobic neutral lipids such as plastoquinone, phylloquinone, α-tocopherol, fatty acid phytyl esters, triacylglycerol, and carotenoids (Kessler et al., 1999; Austin et al., 2006). The first PG-associated protein was discovered in red pepper chromoplasts that contain carotenoid-rich fibrils which are structurally related to PG (Deruère et al., 1994). Hence the members of the newly discovered family of proteins were named fibrillins (FBNs). PG-associated FBNs were identified in many species of plants, algae, and cyanobacteria (Deruère et al., 1994; Pozueta-Romero et al., 1997; Kessler et al., 1999). PGs have functional roles in stress responses, thylakoid breakdown, and chloroplast development (Van Wijk and Kessler, 2017). The proteome of PGs has around 30 proteins, which fall into three categories: FBNs, plastid metabolic proteins [such as tocopherol cyclase (VTE1)], and unknown proteins (Lundquist et al., 2012). Their protein composition suggests that chloroplast PG are heavily involved in prenyllipid and anti-oxidant metabolism protecting thylakoid membranes from ROS-induced damage. In senescing and stressed chloroplasts, the thylakoid membrane is dismantled and chlorophyll is broken down. In these processes, fatty acids (from galactolipids) and phytol (from chlorophyll) are liberated. Membrane-perturbing phytol is esterified with a free fatty acid by PG-localized phytyl ester synthases (Lippold et al., 2012) resulting in non-toxic phytol esters that are deposited inside PG.

In this study, we characterized SOUL4 using a combined biochemical and reverse genetic approach. The data show that SOUL4 is a heme-binding protein. We provide new evidence that SOUL4 is located in chloroplast PG and that it is phosphorylated by an unknown chloroplast kinase.

## Results

### SOUL4 Is Imported Into Chloroplast and Localized at PGs

**Figure 1:**
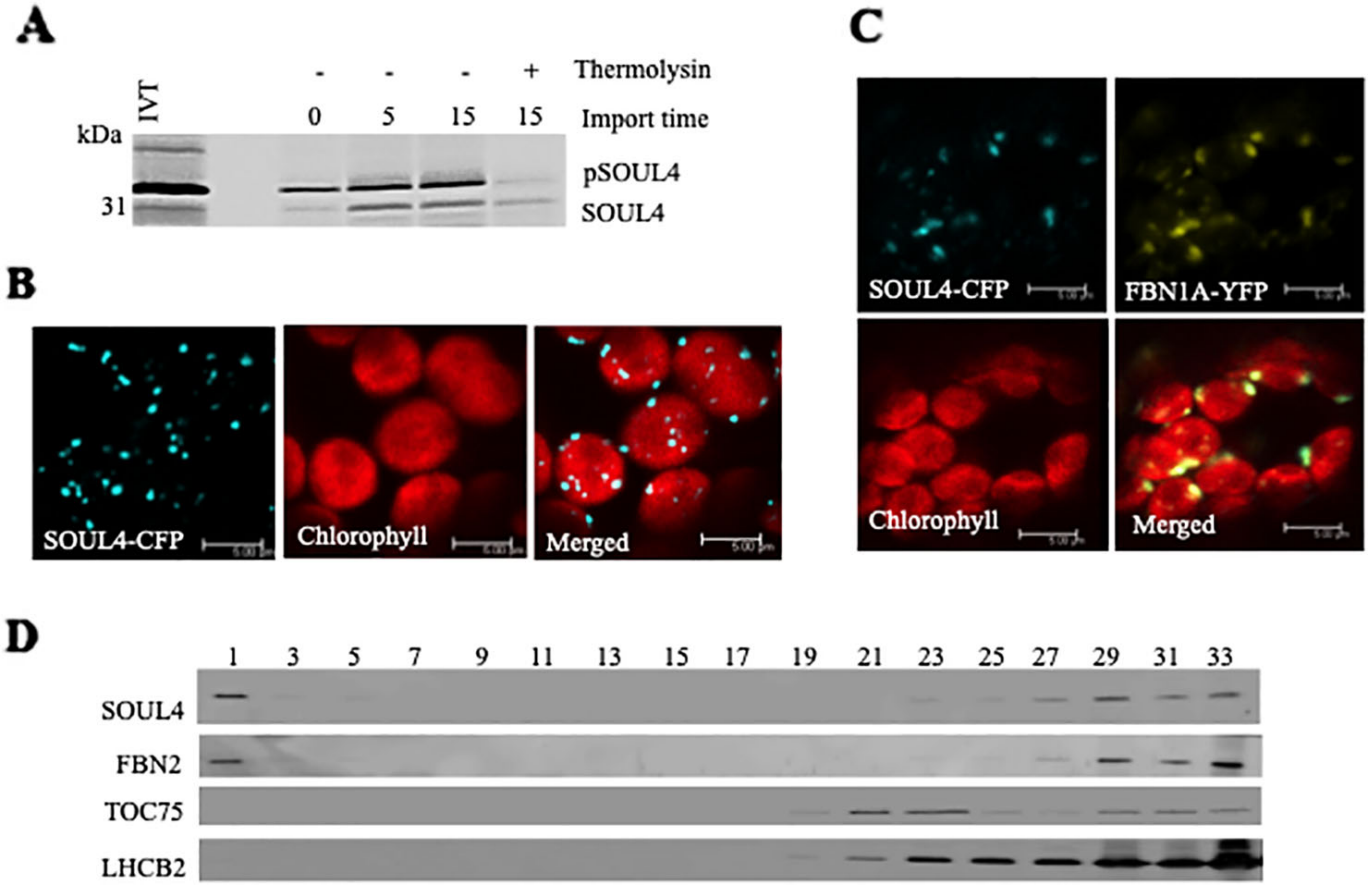
Localization of the SOUL4 heme-binding protein in chloroplast plastoglobules. **(A)** Import of AtSOUL4 preprotein into isolated chloroplasts. *In vitro* synthesized [35S] SOUL4 preprotein (pSOUL4) was incubated with isolated *Arabidopsis* chloroplasts in a time course experiment. After reisolation and thermolysin treatment, the samples were analyzed by SDS-PAGE followed by autoradiography. pSOUL4 (35 kDa) before and mature protein SOUL4 (30 kDa) after thermolysin treatment are indicated. **(B)** Transient expression of SOUL4-CFP in *N. benthamiana* leaves. Fluorescent proteins were visualized by confocal laser scanning microscopy 48 h after agroinfiltration. Chlorophyll, CFP, and merge indicate chlorophyll autofluorescence, CFP fluorescence, and the superposition of both fluorescent signals, respectively. Bar length: 5 μm. **(C)** Colocalization of SOUL4-CFP and FBN1A-YFP. The SOUL4-CFP and FBN1A-YFP were co-expressed transiently in leaves of *N. benthamiana*, and localization was examined by fluorescence microscopy. All images were taken at the same magnification. Chlorophyll: chlorophyll autofluorescence, merge: superposition of YFP and CFP signals (yellow green color). The scale bar represents 5 μm. **(D)** Western blot detection of SOUL4 in chloroplast membrane fractions. After sucrose gradient floatation of total chloroplast membranes, fractions were collected starting from the top of the gradient. Equal volumes of fractions 1–33 were separated by SDS-PAGE and used for immunoblotting with antibodies against SOUL4, FBN2, TOC75 and LHCB2.

Three independent proteomics studies indicate that SOUL4 is mainly present in PGs and may constitute up to 1.8% of the total PG proteome mass (Lundquist et al., 2012). The open reading frame of the SOUL4 cDNA predicts a protein of 309 amino acids including a predicted chloroplast transit peptide of 72 amino acids at the N-terminus as well as the SOUL heme–binding motif. We further analyzed whether the SOUL4 protein was imported into the chloroplast, whether its predicted transit peptide was processed, and whether SOUL4 is targeted to PGs. The SOUL4 preprotein was synthesized in a coupled transcription/translation system in the presence of [^35^S]methionine and incubated with isolated *Arabidopsis* chloroplasts *in vitro*. Two major radioactively labeled proteins were detected: one with an apparent molecular mass (MM) of around 35 kDa corresponding to the expected MM of the *in vitro* synthesized SOUL4 preprotein and the second band with an apparent MM of around 30 kDa corresponding to the mature form of SOUL4 (**Figure 1A**) (Venkatasalam, 2012). After reisolation and treatment of the chloroplasts with thermolysin, most of the 35 kDa polypeptide was digested, whereas the 30 kDa protein was largely protected against proteolytic attack. This shows that the SOUL4 preprotein was imported into the chloroplast and processed to its mature form. To address targeting of SOUL4 to PG, we engineered a construct encoding a SOUL4-CFP fusion under the control of the cauliflower mosaic virus (CaMV) 35S promoter. The SOUL4-CFP construct was transformed into *Nicotiana benthamiana* leaves by agroinfiltration and analyzed by confocal laser scanning microscopy. The SOUL4-CFP fluorescence was present in punctate structures reminiscent of PG (**Figure 1B**), but was detected neither in the thylakoid nor the envelope membranes (Venkatasalam, 2012). To determine whether SOUL4 colocalized with the PG marker FBN1A, the two proteins were co-expressed as CFP and YFP fusion proteins in *N. benthamiana* leaves by agroinfiltration. In this experiment, SOUL4-CFP as well as FBN1A-YFP fluorescence was present in the punctate structures inside chloroplasts. Both the merge of the independent fluorescence images and the pixel intensity analysis showed a signal overlap, indicating colocalization of SOUL4 and FBN1A in PGs (**Figure 1C**) (Venkatasalam, 2012). In addition, overexpression of SOUL4-YFP in stable transgenic plants gave punctate fluorescence pattern inside chloroplasts (see **Supplementary Figure 1**). To obtain physical evidence of PG association, total membranes of isolated chloroplasts were fractionated on a sucrose gradient. This resulted in the separation of membrane fractions corresponding to broad and partially overlapping peaks of marker proteins by immunoblotting analysis. Antibodies against LHCB2 gave strong signals in fractions 23–33 (**Figure 1D**), indicating the presence of thylakoid membranes in the high density sucrose fractions. TOC75, a protein import channel of the outer envelope membrane, was mainly detected in fractions 21–23 (**Figure 1D**). SOUL4 co-distributed with FBN2 in the lowest density fraction (**Figure 1D**) (Venkatasalam, 2012). Presence of SOUL4 and FBN2 in the higher density fractions is in part due to incomplete separation of PG from thylakoid membranes and in part to bona fide partitioning between PG and thylakoid membranes (Lundquist et al., 2012).

### The SOUL4 Protein Binds Heme *In Vitro*

**Figure 2:**
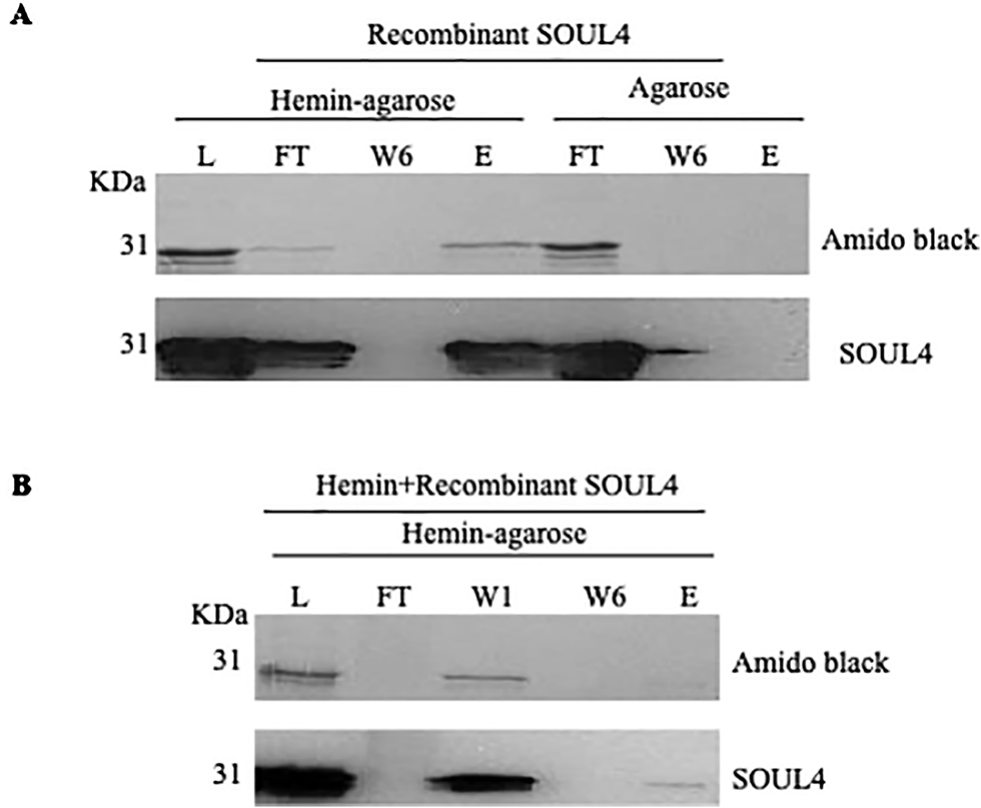
*In vitro* heme-binding by mature SOUL4. **(A)** Binding of recombinant mature SOUL4 to hemin-agarose or agarose alone, as a negative control. The load (L), flow-through (FT), wash 6 (W6), and eluted (E) fractions were analyzed by SDS-PAGE, transferred to a nitrocellulose membrane, and stained with Amido black (upper panel). Antibodies against SOUL4 were used for immunoblotting, as indicated (lower panels). **(B)** SOUL4 binding to hemin-agarose in the presence of 10 mM added soluble hemin. The load (L), wash 1, wash 6 (W1, W6), and eluted (E) fractions were analyzed by SDS-PAGE, transferred to a nitrocellulose membrane, and stained with Amido black (upper panel). Antibodies against AtSOUL4 were used for immunoblotting as indicated (lower panels).

Structural studies of SOUL protein revealed a novel hydrophobic cleft flanked by an α-helix and the β8–β9 loop that was essential for heme-binding (Dias et al., 2006). All five *Arabidopsis thaliana* homologs have this novel hydrophobic cleft for heme-binding as it has been already experimentally demonstrated for AtSOUL1 and AtSOUL2 (Takahashi et al., 2008). Mature SOUL4 was expressed in *Escherichia coli*, purified as a soluble protein, and confirmed by immunoblotting. To investigate heme-binding properties, the purified recombinant SOUL4 was incubated with hemin-agarose and agarose alone as a negative control. Input, flow-through, last wash, and eluate fractions were analyzed by immunoblotting. SOUL4 bound to hemin-agarose and was detected in the eluate fraction (**Figure 2A**, left panel, lane E), while it was not found in eluate fraction of the agarose alone (**Figure 2A**, right panel, lane E) (Venkatasalam, 2012). To exclude non-specific binding to hemin-agarose, a heme competition assay was carried out. SOUL4 (100 μM) was incubated with 10 mM hemin. After the incubation, the reaction mixture was applied to the hemin-agarose and subsequently washed and eluted. The load, flow-through, and wash fractions as well as the eluate were separated by SDS-PAGE followed by immunoblot analysis. The first wash removed most of the non-bound SOUL4, and the eluate contained very little SOUL4, demonstrating that SOUL4 binding to hemin-agarose was strongly impaired after incubation with 10 mM hemin (**Figure 2B**) (Venkatasalam, 2012). Together, the data indicate that SOUL4 specifically binds heme *in vitro*.

### Characterization of T-DNA Insertion Mutant of *SOUL4*

**Figure 3:**
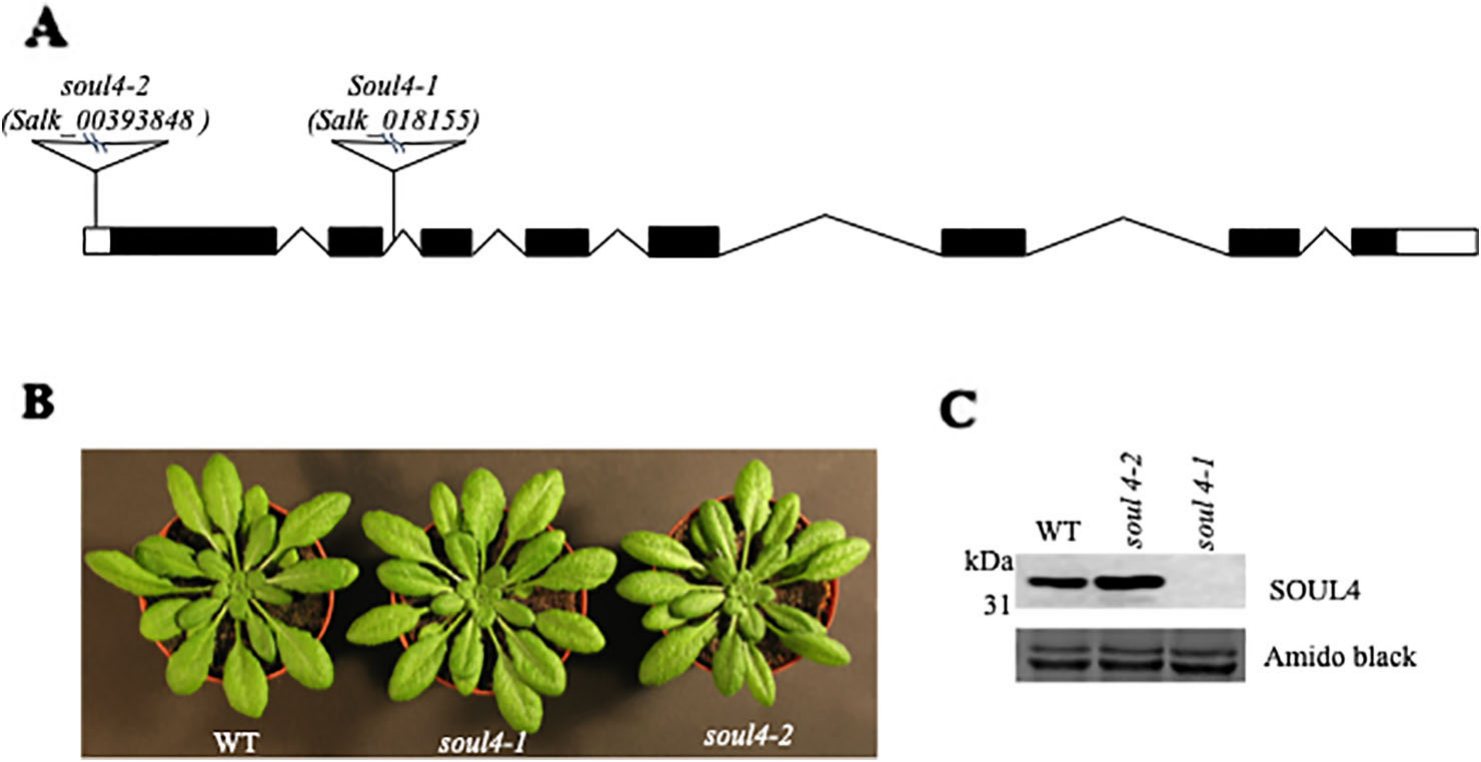
Characterization of *soul4* mutant in *Arabidopsis*. **(A)** Scheme showing the position of the T-DNA insertion in the *SOUL4* gene. Introns are represented by black boxes and exons by black lines; 5'- and 3'-untranslated regions are shown as empty boxes. **(B)** Four-week-old wild type (Col-0), *soul4-1*, and *soul4-2* plants were grown on soil. **(C)** Western blot analyses of total protein extracts for WT, *soul4-2*, and *soul4-1* mutant were separated by SDS-PAGE, transferred to nitrocellulose membrane, and stained with Amido black for a loading control (lower panel). Antibody against SOUL4 was used for immunoblotting, as indicated (upper panel).

**Figure 4:**
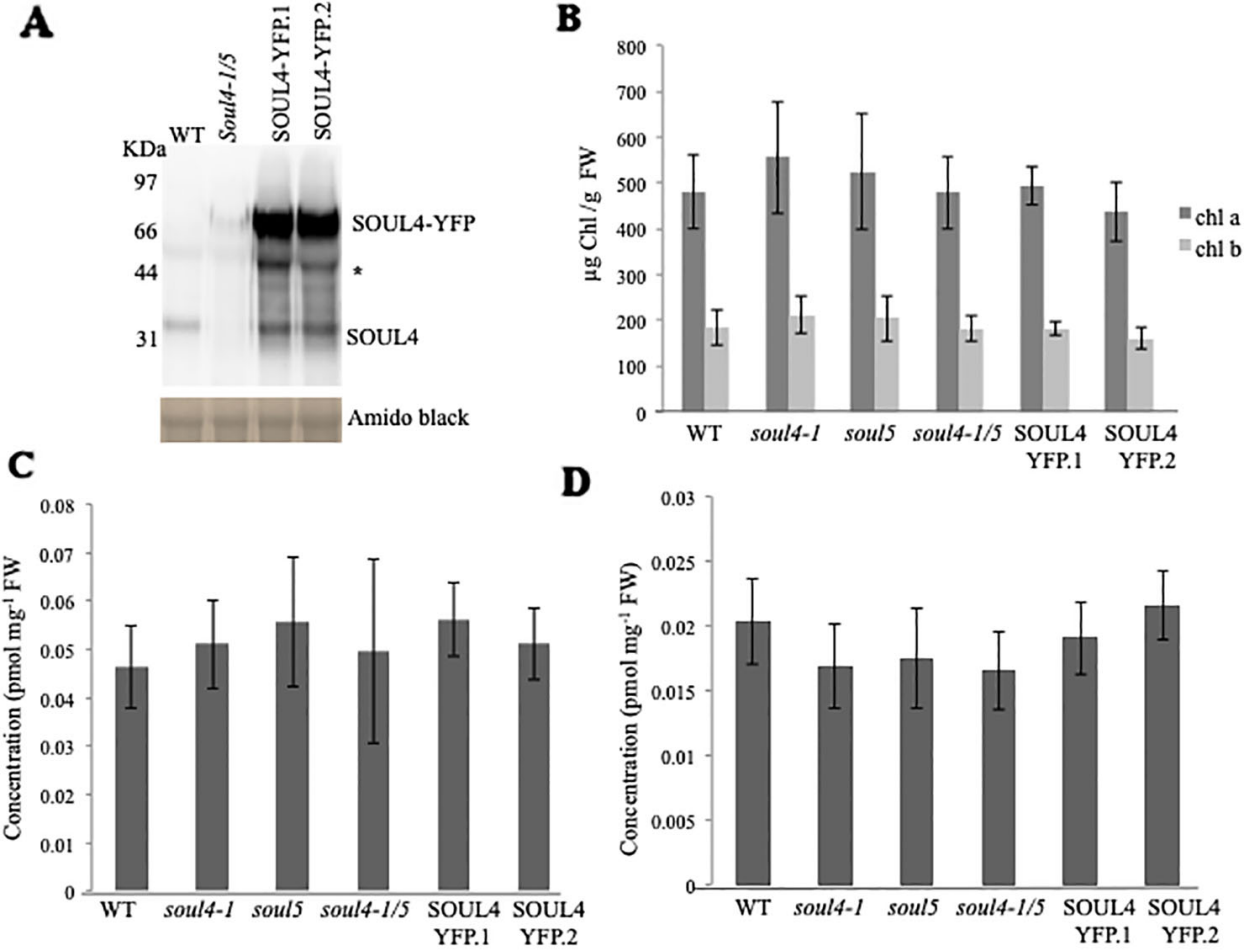
Comparison of tetrapyrroles and chlorophyll pigment levels in *soul* mutants and SOUL4-YFP overexpressing lines. **(A)** Western blot analyses of total protein extracts for WT, *soul4-1/5*, and SOUL4-YFP overexpressing lines SOUL4-YFP.1 and SOUL4-YFP.2 were separated by SDS-PAGE, transferred to a nitrocellulose membrane, and stained with Amido black for a loading control (lower panel). Antibodies against SOUL4 were used for immunoblotting, as indicated (upper panel) and * indicated as a non-specific band. Chlorophyll a, b **(B)**, Mg-protoporphyrin IX **(C)**, and con-covalently bound heme **(D)** contents measured from wild type, *soul4-1*, *soul5*, *soul4-1/5*, and SOUL4-YFP overexpressing lines SOUL4-YFP.1 and SOUL4-YFP.2. The samples were extracted from 3-week-old *Arabidopsis* seedlings grown on soil under short-day condition. Data are averages of four biological replicates (± SD).

We isolated *Arabidopsis* SOUL4 T-DNA insertion lines. The *soul4-1* insertion line contained T-DNA insertions in intron 2 and *soul4-2* insertion line contained T-DNA insertions in **5**′ untranslated region (**5**′ UTRs) (**Figure 3A**) (Venkatasalam, 2012). Homozygous lines were obtained by selfing and verified by segregation analysis and genotyping. The SOUL4 protein was not immunologically detected in total leaf extracts of *soul4-1* confirming a knockout genotype of nature of the T-DNA insertion (**Figure 3C**) (Venkatasalam, 2012). Under standard growth conditions on soil, homozygous *soul4-1* and *soul4-2* grew normally and exhibited a wild-type phenotype (**Figure 3B**) (Venkatasalam, 2012). Earlier studies had shown that a number of PG proteins [e.g. VTE1 and NAD(P)H dehydrogenase C1 (NDC1)] are involved in prenylquinone metabolism (Vidi et al., 2006; Eugeni Piller et al., 2011). To determine whether SOUL4 plays a role in prenylquinone metabolism, untargeted analyses as well as targeted prenylquinone profiling were carried out. The clustering of the *soul4-1* and WT-derived metabolomes revealed no differences by principal component analysis of either untargeted lipidomics or targeted prenylquinone profiling data (data not shown) indicating that the two genotypes have largely identical metabolomes under standard growth conditions. Phylogenetic analysis of *Arabidopsis* SOUL proteins revealed that SOUL5 was a close homolog of SOUL4 (see **Supplementary Figure 2**) and moreover, SOUL5 is also localized in the chloroplast. To investigate the role of two chloroplast SOUL proteins in tetrapyrrole accumulation, we also isolated the *soul5* and *soul4-1/5* mutants (see **Supplementary Figure 3**). In higher plants, the chlorophyll- and heme-synthesizing pathways play essential roles in cellular and organellar function. We measured the content of chlorophyll *a* and *b*, Mg-protoporphyrin IX, and heme from wild type, *soul4-1, soul5*, *soul4-1/5*, and SOUL4-YFP overexpressing lines (SOUL4-YFP1 and SOUL4-YFP2) under moderate light (120 µmol m^−2^ s^−1^). The comparative analysis revealed no significant differences in the amount of the tetrapyrrole intermediates and end product between WT, *soul* mutant, and a SOUL4 overexpressor line (**Figure 4**) (Venkatasalam, 2012).

### Casein Kinase II and Chloroplast Stroma Phosphorylate SOUL4 Protein *In Vitro*

**Figure 5:**
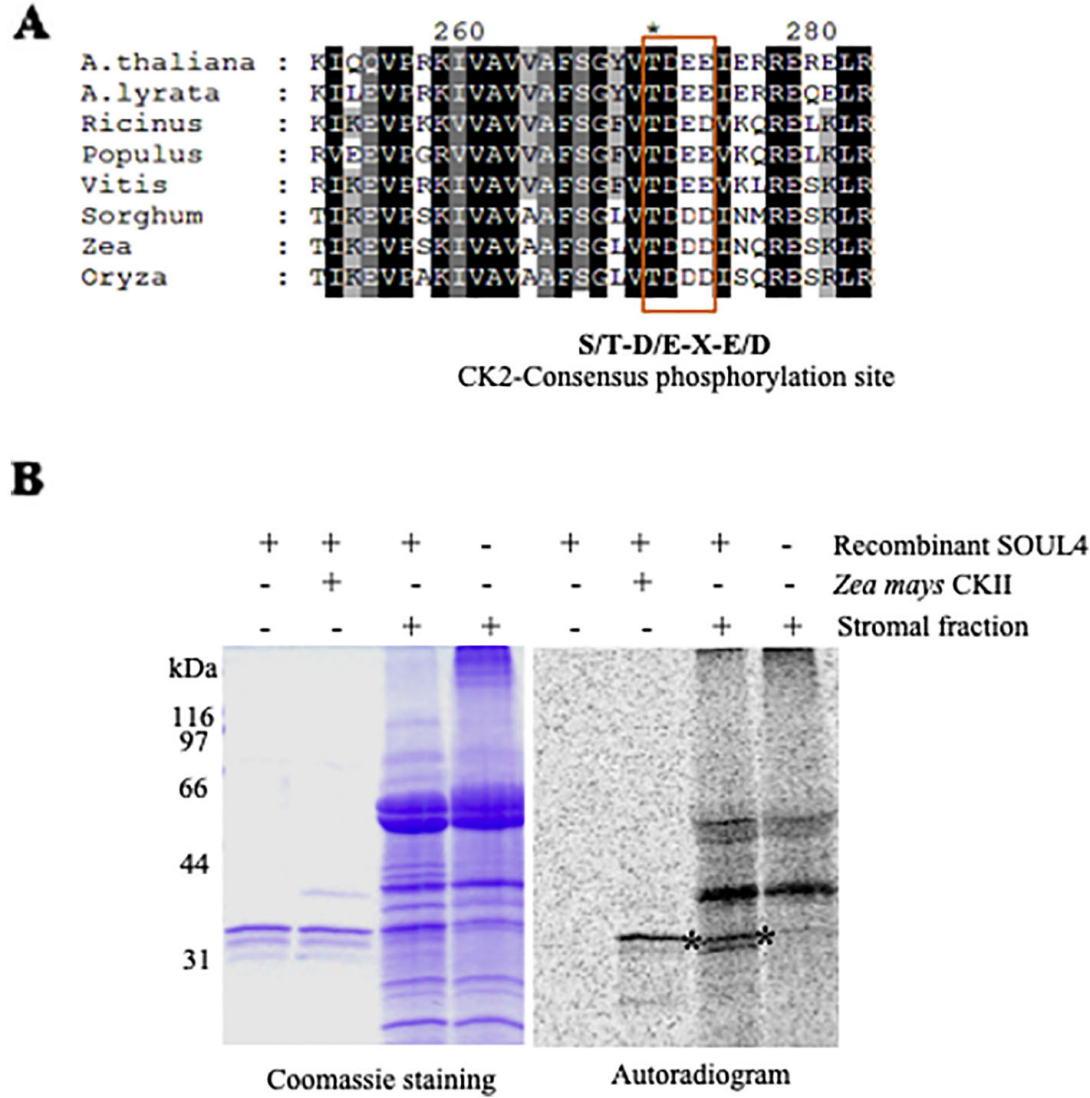
Phosphorylation of recombinant SOUL4 heme-binding protein by CK2 and stromal fractions **(A)** Alignment of SOUL4 with its homologs of other species indicating the predicted CKII phosphorylation site. SOUL4 from *Arabidopsis thaliana* NP_187624.1, *A. lyrata* XP_002882654.1, *Ricinus communis* XP_002528755.1, *Populus trichocarpa* XP_002323500.1, *Vitis vinifera* XP_002282544.1, *Sorghum bicolor* XP_002452205.1, *Zea mays* NP_001147650.1, *Oryza sativa* NP_001047029.1 aligned by ClustalW. The C-terminal conserved casein kinase II (CKII) motif is highlighted and boxed. **(B)** Purified recombinant AtSOUL4 (3 μg) was incubated without (lane 1) or with (lane 2) recombinant *Z. mays* CKII α and [γ33P] ATP as the phosphate donor. In addition, recombinant AtSOUL4 was incubated with (lane 3) or without (lane 4) isolated *Arabidopsis* stromal fraction. The samples were separated by SDS-PAGE followed by Coomassie blue staining (left panel) and phosphorimager analysis (right panel). The asterisk symbol (*) indicated as a specific band of phosphorylated AtSOUL4.

SOUL3 phosphorylation has been identified in the eyespot of *C. reinhardtii* (Wagner et al., 2008). We therefore examined SOUL4 phosphorylation in *Arabidopsis*. The C-terminus of SOUL4 has a conserved casein kinase II (CKII) target consensus sequence (S/T-D/E-X-E/D) (**Figure 5A**) (Venkatasalam, 2012) and may therefore be a target of CKII like kinase. One of the five *Arabidopsis* CKII α subunits has been localized in the chloroplast stroma (Salinas et al., 2006). To determine whether recombinant SOUL4 is an *in vitro* substrate of CKII, we incubated recombinant SOUL4 with (**Figure 5B**, lane 2) or without (**Figure 5B**, lane 1) the recombinant maize CKII α subunit in a phosphorylation assay. To test whether SOUL4 is a potential substrate of the stromal CKII or other stromal kinases, the *in vitro* phosphorylation assay using recombinant SOUL4 was carried out with (**Figure 5B**, lane 3) or without (**Figure 5B**, lane 4) *Arabidopsis* stromal fraction (Venkatasalam, 2012). All the samples were incubated in the presence of [γ^33^P] ATP. The samples were separated by SDS-PAGE followed by phosphorimager analysis, revealing phosphorylation of SOUL4 both by recombinant maize CKII and stromal fraction.

## Discussion

SOUL4 was synthesized as a 35 kDa protein *in vitro* and converted to a 30 kDa, protease-resistant protein in an *in vitro* import assay into isolated chloroplasts. These results indicate that SOUL4 is synthesized as a cytosolic preprotein with a transit peptide (N-terminal chloroplast targeting signal) that is cleaved upon import into chloroplasts. However, this does not provide any evidence regarding the suborganellar localization. Transient expression of a SOUL4-CFP fusion in tobacco leaves resulted in punctate fluorescence signals inside the chloroplast, indicative of a PG sublocalization. This was corroborated by colocalization with a well-accepted PG marker, FBN1a-YFP, and by co-distribution of SOUL4 with FBN2 by membrane fractionation on a sucrose gradient. *Arabidopsis* SOUL1, SOUL2, and SOUL5 homologs of SOUL4 are known examples of heme-binding proteins with well-characterized biochemical properties (Takahashi et al., 2008; Lee et al., 2012). Purified recombinant SOUL4 (expressed without the transit peptide) bound to hemin-agarose *in vitro*, and the binding was competed by preincubation of SOUL4 with free heme. This, in addition to the predicted tetrapyrrole-binding cleft, are strong indications that SOUL4 also acts as heme- or tetrapyrrole-binding protein *in vivo*. A number of known chloroplast metabolic enzymes and previously unclassified proteins are known to function in PG lipid metabolism [e.g. the VTE1 (Vidi et al., 2006), NDC1 (Eugeni Piller et al., 2011), and PES1 and 2 (Lippold et al., 2012)], and therefore, the potential role of SOUL4 in PG lipid metabolism was investigated using prenyllipid analysis. Under standard growth conditions, no significant differences in the lipid profiles of *soul4-1* and WT were observed (Venkatasalam, 2012). However, this does not exclude that minor lipid components may have escaped detection or that major changes may be induced under stress conditions. SOUL4 has a conserved CKII target consensus sequence (S/T-D/E-X-E/D), and *C. reinhardtii* SOUL3 was present in the phosphoproteome of the eyespot that also contains other proteins conserved from PG (Wagner et al., 2008). A chloroplastic stromal CKII α subunit has been reported in mustard (Baginsky et al., 1999; Ogrzewalla et al., 2002) as well as *Arabidopsis* (Salinas et al., 2006). Chloroplastic CKII has a wide range of predicted substrates (Baginsky and Gruissem, 2009). Recombinant, purified SOUL4 was readily phosphorylated *in vitro* by maize CKII α subunit as well as an isolated stromal extract. The isolated extract is known to contain stromal CKII but most likely also other kinases. Therefore, it cannot be excluded that unknown stromal kinases other than CKII are able to phosphorylate SOUL4 *in vitro*. It appears likely that phosphorylation is a regulatory mechanism in SOUL4, but in view of the absence of a well-characterized function, its purpose remains unclear. The phenotypic analysis of the *soul* mutant and SOUL4 overexpressing lines grown on soil under standard conditions did not yield an apparent phenotype when compared to wild type. An exhaustive tetrapyrrole analysis suggested that under standard growth conditions, there was no significant difference in tetrapyrroles between WT, *soul4-1*, and SOUL4 overexpressing lines. Our results suggest that SOUL4 acts as heme-binding protein and may contribute to a pool of free heme molecules inside the chloroplast. The entire content of the covalently bound (mainly a- and c-type) and non-covalently bound (b-type) heme can currently not be determined. Methods are available to determine the non-covalently bound heme and more recently the free or labile heme portion (Weinstein and Beale, 1983; Masuda and Takahashi, 2006; Richter et al., 2019). The latter pool of heme is certainly only a subfraction of the non-covalently bound heme, but could likely include the heme portion bound to SOUL isoforms. It will be of future interest to determine heme-binding affinities of SOUL proteins and the dynamic of heme exchange of this group of proteins.

## Materials and Methods

### Plant Material and Growth

Wild-type and mutant *A. thaliana* plants, ecotype Colombia, were used in all experiments. The *soul4-1* (Salk_018155), *soul4-2* (Salk_00393848), *soul5* (SAIL_215_CO2), double mutant was generated by crossing *soul4-1* and *soul5* homozygous plants followed by genotyping (see **Supplementary Table 1**). Plants were grown either on soil under long-day conditions [16 h light (120 μmol m^−2^ s^−1^), 8 h dark, 21°C, 70% relative humidity] or on Murashige and Skoog (MS) medium under short-day conditions [8 h light (120 μmol m^−2^ s^−1^), 16 h dark, 21°C, 70% relative humidity).

### Localization Studies by Fluorescent Marker

The full length *Arabidopsis SOUL4* cDNA was amplified using the SOUL4-F and SOUL4-R primers (see **Supplementary Table 1**) flanked by NcoI restriction sites and ligated into the NcoI site of the pCL62 vector, encoding a C-terminally CFP-tagged fusion protein. The pCL61 vector, encoding for the C-terminally YFP-tagged fusion protein of FBN1A, was used as PG marker. Both constructs were transformed into *A. tumefaciens* strain C58Ci. The SOUL4-CFP construct and the combination of SOUL4-CFP, FBN1A-YFP constructs were co-infiltrated into leaves of 2- to 3-week-old *N. benthamiana* plants. Infiltrated leaves were analyzed after 2 days under a Leica TCS SL confocal microscope. CFP and YFP were detected sequentially using 458 and 514 nm laser lines, as well as 460–510 and 520–588 nm detection windows, from a LEICA SP2 AOBS microscope. Chlorophyll autofluorescence was monitored using either 594 nm or TRITC (568 nm) excitation wavelengths. Images were captured and analyzed using LCS lite software (Leica).

### Chloroplast Isolation and *In Vitro* Import Reactions

*Arabidopsis* plants were grown on MS medium as described. Intact chloroplasts were isolated according to Fitzpatrick and Keegstra, 2001. Full length *SOUL4* cDNA was amplified using the SOUL4-F and SOUL4-R primers (see **Supplementary Table 1**) flanked by *NcoI* restriction sites and ligated into the *NcoI* site of the pET21d vector. The pET21d-SOUL4 construct contains a T7 promoter upstream of the start codon used for *in vitro* protein synthesis using the TNT^®^ T7 Quick Coupled Transcription/Translation System (Promega Corporation, Madison WI, USA) as recommended by the supplier. Chloroplasts corresponding to 25 μg chlorophyll were used for import assays. The import experiment of *in vitro* synthesized proteins into intact isolated chloroplasts has been described previously (Agne et al., 2009).

### Expression and Purification of Recombinant Proteins

*SOUL4* cDNA without the sequence encoding a transit peptide (72–309 amino acid) was amplified using the SOUL4-F(-TP) and SOUL4-R primers (see **Supplementary Table 1**) flanked by NcoI restriction sites and ligated into NcoI site of the pET21d vector, encoding for a C-terminally His_6_-tagged fusion protein (pET21d-SOUL4-HIS). Mature SOUL4 protein was overexpressed in *E. coli* BL21 (DE3) transformed with the expression vector pET21d-SOUL4-HIS. Expression was induced using 0.4 mM isopropyl-β-D-thiogalactopyranoside at 37°C for 3 h. Bacterial pellets were lysed by sonication in 50 mM Tris-HCl pH 8, 150 mM NaCl, and 5 mM imidazol followed by centrifugation for 30 min at 14,000 rpm (Sorvall, SS-34). The supernatant was filtered through a 0.45 μM nitrocellulose filter, and the mature SOUL4-HIS protein was purified from the supernatant fraction by nickel–nitrilotriacetic acid agarose affinity chromatography. Eluates were dialyzed against phosphate buffered saline (PBS). Glycerol (10%) was added to the purified SOUL4-HIS protein and stored at −80°C.

### *In Vitro* Heme-Binding Assay

The *in vitro* heme-binding assay was carried out as described (Vanhee et al., 2011). Recombinant SOUL4 protein (100 µM) in PBS with glycerol was incubated with hemin-agarose (Sigma-Aldrich) or agarose beads for 1 h on a rotating wheel. The flow-through was collected and the beads washed with PBS buffer and eluted with SDS-PAGE–loading buffer. For the heme competition assay, 100 µM of recombinant mature SOUL4 protein in volume of 80 µl was pre-incubated with 20 µl of 10 mM hemin solution for 1 h before incubation with the hemin-agarose. The flow-through was collected and the beads washed with PBS buffer and eluted with SDS-PAGE–loading buffer. The input, flow-through, last wash, and elution were analyzed by SDS-PAGE and Western blot.

### Porphyrin and Heme Analysis

Porphyrins were extracted and analyzed as described previously (Papenbrock et al., 2000). The non-covalently bound heme was extracted by acidic acetone containing 5% HCl, transferred to diethyl ether, concentrated, and washed on a DEAE-Sepharose column. Measurement of heme content has been described previously (Castelfranco and Jones, 1975).

### Plant Transformation and Transgenic Lines

The full length *Arabidopsis* SOUL4 cDNA was amplified using the SOUL4-AttB1 and SOUL4-AttB2 primers and ligated into the pENTR221 vector using BP clonase (Invitrogen) and inserted into the binary vector pEarleyGate101 using the LR clonase (Invitrogen), resulting in a vector coding for the SOUL4-YFP fusion under the control of the 35S promoter. *A. thaliana* plants were transformed using the floral dip method as described (Clough and Bent, 1998).

### Protein Extraction and Immunoblotting

Leaves were homogenized in 250 µl of Rensink buffer [100 mM NaCl, 50 mM Tris-HCl, pH 7.5, 1% (v/v) Triton X-100, and 10 mM β-mercaptoethanol] supplemented with protease inhibitor mixture (Sigma P9599) and centrifuged at 20,000×g for 5 min. Protein was quantified in the supernatant using the Bradford method. Proteins were concentrated by chloroform–methanol precipitation, separated by SDS-PAGE, and blotted onto nitrocellulose membrane. After protein transfer, the nitrocellulose membranes were stained with Amido black. Polyclonal anti-SOUL4 and anti-FBN2 were obtained from rabbits immunized with recombinant SOUL4-HIS and FBN2-HIS protein (Eurogentec, Seraing, Belgium). Antibodies were further affinity-purified using recombinant protein coupled to Affi-gel10 (Bio-Rad Laboratories) according to the supplier's instructions. To probe the blots, primary antibodies recognizing SOUL4, FBN2, TOC75, and LHCB2 (Agrisera) were used.

### Phosphorylation Assays

Three micrograms of recombinant SOUL4 protein were incubated with or without 37.5 units of recombinant maize (*Zea mays*) CK2 a-subunit (Biaffin) and with or without stromal fractions in the presence of 25 mM Tris-HCl, pH 8.0, 5 mM MgCl2, 1 mM dithiothreitol (DTT), 50 mM ATP, and 1 µCi of [γ-33P] ATP for 30 min at 25°C. Reactions were stopped by diluting in ice-cold buffer followed by chloroform–methanol precipitation. The samples were separated by SDS-PAGE and examined by autoradiography.

### Chloroplast Membrane Fractionation and PG Isolation

Chloroplasts membranes were isolated as previously reported (Vidi et al., 2006). Total membranes, corresponding to 10 mg of chlorophyll, were sedimented at 100,000×g and resuspended in 2 ml 45% sucrose in TE buffer. Membranes were overlaid with a linear sucrose gradient (5–45% sucrose) and centrifuged for 17 h at 100,000×g and 4°C (SW41Ti rotor, Beckman). Fractions (1 ml) were collected starting from the top of the gradient and used for Western blotting.

## Data Availability Statement

The datasets generated for this study are available on request to the corresponding author.

## Author Contributions

VS and FK designed the experiments. BG contributed the tetrapyrrole and chlorophyll analyses. VS performed all experiments. VS, FK, and BG analyzed the data, and VS wrote the manuscript with the help of FK and BG.

## Funding

This work was supported by the Swiss National Science Foundation (SNSF) grants 31003A_156998 and 31003A_176191. Work in BG's laboratory was partially supported by the Deutsche Forschungsgemeinschaft (Subproject C04 of the DFG SFB/TR175).

## Conflict of Interest

The authors declare that the research was conducted in the absence of any commercial or financial relationships that could be construed as a potential conflict of interest.
