# Supplementary material for: Characterization of a Plastoglobule-Localized SOUL4 Heme-Binding Protein in Arabidopsis thaliana
Source: Front Plant Sci. 2020 Jan 31;11:2. doi: 10.3389/fpls.2020.00002 (PMC7006542; doi:10.3389/fpls.2020.00002)
Supplement: Supplementary file 1 [file DataSheet_1.docx]

Supplementary Material

**Characterization of a plastoglobule-localized SOUL4 heme-binding protein in *Arabidopsis thaliana***

Venkatasalam Shanmugabalaji ^1*^, Bernhard Grimm^2^, Felix Kessler ^1^

*** Correspondence:**

Venkatasalam Shanmugabalaji

Email: shanmugabalaji.venkatasalam@unine.ch

**
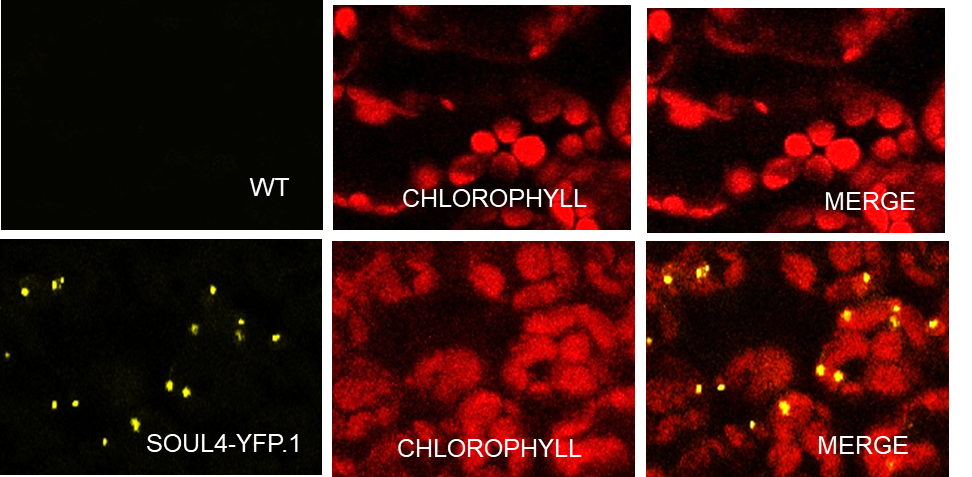
**

**Supplementary Figure S1. Localisation of the SOUL4 heme-binding protein in transgenic Arabidopsis**

Detection of YFP fluorescence in the stably transformed AtSOUL4-YFP.1 line by confocal laser-scanning microscopy using a wild type control (WT). Yellow fluorescence (YFP), red fluorescence (chlorophyll) and the overlay of the two are shown for the transgenic AtSOUL4-YFP.1 line and the wild type (WT) control.

**
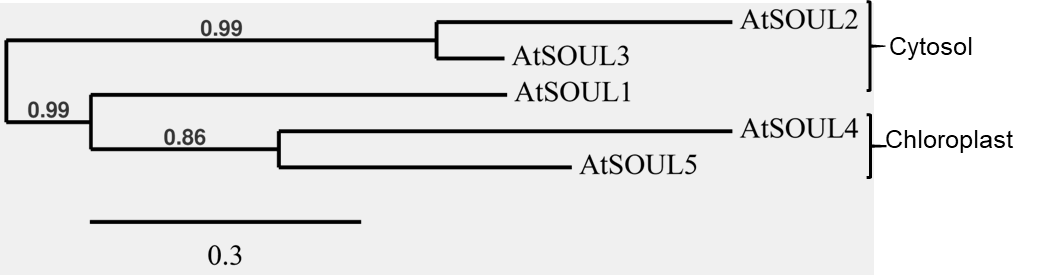
**

**Supplementary Figure S2. Phylogenetic analysis of SOUL proteins in Arabidopsis.**

The neighbour-joining phylogenetic tree was constructed by using TreeDyn 198.3 after CLUSTALW alignment of the amino acid sequences of Arabidopsis SOUL binding proteins.

**A**

**B**

(*SOUL5*)

(*T-DNA*)


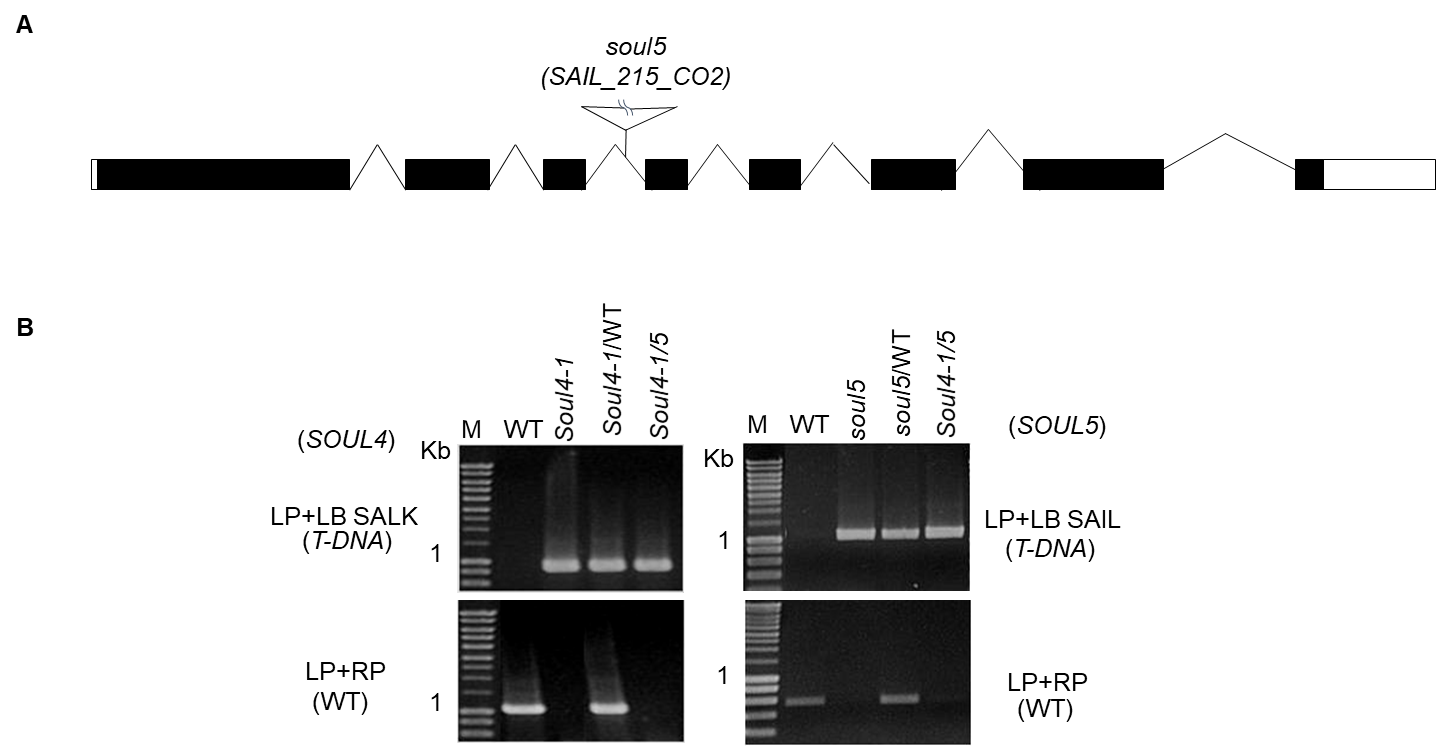


**Supplementary Figure S3. Isolation of *soul5 and soul4-1/5* double mutants**

(A) Scheme showing the position of the T-DNA insertion in the *soul5* gene. Introns are represented by black boxes and exons by black lines; 5'- and 3'-untranslated regions are shown as empty boxes. (B) PCR analysis of genomic DNA from heterozygous and homozygous of *soul4-1, soul5* and double knockout *soul4-1/soul5*. The absence of the wild-type PCR product indicates the homozygous nature of an allele; a wild-type control (WT) is included. The gene specific primers indicated in the top (*SOUL4* and *SOUL5*). Lane M shows molecular size markers.

**Supplementary Table 1**

List of primers used for genotyping and cloning

| Primer name | sequence |
| --- | --- |
| SOUL4F | cgaagagccatggtgatgatcagctcc |
| SOUL4R | gggccatggcttctttgttttcaacttcg |
| SOUL4F(-TP) | ccttgctcccatggctcaagcttcctctg |
| Soul4 AttB F | ggg gac aag ttt gta caa aaa agc agg cta tgg tca tga tca gct cc |
| Soul4 AttB R | ggg gac cac ttt gta caa gaa agc tgg gtg ggc ttc ttt gtt ttc aac ttc g |
| SOUL4-1 LP | tttgagggcatgacaaaagac |
| SOUL4-1 RP | atgcccaattacttactccgg |
| SOUL4-2 LP | TCG ACCCTTGTA AAGTTG TGC |
| SOUL4-2 LP | CTTGGGAAACACATATTTCGC |
| SOUL5- LP | CTTTGG AGTTTCCCAAGTCTGGCG |
| SOUL5- rP | GGTCTTGTTGATGTTTTTAAGCAG |
| LBpROK2 SALK | TGGACTCTTGTTCCAAACTG |
| LBCSA110 SAIL | GCCTTTTCAGAAATGGATAAATAGCC |
